# Obituary. Dr. Charles B. Coventry

**Published:** 1875-03

**Authors:** 


### OBITUARY.

DR. CHARLES B. COVENTRY, of Utica, N. Y., Emeritus Professor of Physiology and Medical Jurisprudence in the Buffalo Medical College, died at his residence in the latter part of February. Dr. Coventry was born in Deerfield, N. Y., in April, 1801, and was therefore, at the time of his death neary 74 years of age. He graduated as Doctor of Medicine in 1825, at the College of Physicians and Surgeons of Western New York, then situated at Fairfield, N. Y. He at once entered upon the practice of his profession in company with his father, Dr. Alexander Coventry. During the course of his life he held many honorable positions, and made several contributions of value to the literature of medicine. In 1828 he was appointed Lecturer on Materia Medica in the Berkshire Medical College, which position he resigned at the end of three years. In 1839 he assumed the chair of Materia Medica and Obsterics in the Geneva Medical College, afterwards changing to that of Obstetrics and Medical Jurisprudence. In 1840 he was called to the position in the Buffalo Medical College, which he has held ever since, though of late only as Emeritus Professor.

At a meeting of the Faculty of the Medical Department of the University of Buffalo, held March 2d, 1875, the following resolutions were passed on the announcement of the death of Prof. Chas. B. Coventry, of Utica, N. Y.

*Resolved*, That we hear with great regret of the death of our former colleague, who was associated with us at the founding of the Medical Department of the University of Buffalo, and for several succeeding years;

That we desire to give our testimony to his conscientious discharge of his duties as a teacher, and to his thorough kindness, amiability, and uprightness of character, which through all our intercourse endeared him personally to each one of us.

To his relatives and friends we offer our sincere sympathy in their great affliction.

